# The Molecular Landscape Influencing Prognoses of Epithelial Ovarian Cancer

**DOI:** 10.3390/biom11070998

**Published:** 2021-07-07

**Authors:** Chao-Lien Liu, Ray-Hwang Yuan, Tsui-Lien Mao

**Affiliations:** 1 School of Medical Laboratory Science and Biotechnology, College of Medical Science and Technology, Taipei Medical University, Taipei 11031, Taiwan; chaolien@tmu.edu.tw; 2 PhD Program in Medical Biotechnology, College of Medical Science and Technology, Taipei Medical University, Taipei 11031, Taiwan; 3 Department of Surgery, National Taiwan University Hospital, Taipei 10002, Taiwan; d83409009@ntu.edu.tw; 4 Department of Surgery, College of Medicine, National Taiwan University, Taipei 10002, Taiwan; 5 Department of Pathology, College of Medicine, National Taiwan University, Taipei 10002, Taiwan; 6 Department of Pathology, National Taiwan University Hospital, Taipei 10002, Taiwan

**Keywords:** epithelial ovarian cancer (EOC), mutation, biomarker, genome/epigenome, therapeutic targets, personalized medicine

## Abstract

Epithelial ovarian cancer (EOC) is one of the major increasing lethal malignancies of the gynecological tract, mostly due to delayed diagnosis and chemoresistance, as well as its very heterogeneous genetic makeup. Application of high-throughput molecular technologies, gene expression microarrays, and powerful preclinical models has provided a deeper understanding of the molecular characteristics of EOC. Therefore, molecular markers have become a potent tool in EOC management, including prediction of aggressiveness, prognosis, and recurrence, and identification of novel therapeutic targets. In addition, biomarkers derived from genomic/epigenomic alterations (e.g., gene mutations, copy number aberrations, and DNA methylation) enable targeted treatment of affected signaling pathways in advanced EOC, thereby improving the effectiveness of traditional treatments. This review outlines the molecular landscape and discusses the impacts of biomarkers on the detection, diagnosis, surveillance, and therapeutic targets of EOC. These findings focus on the necessity to translate these potential biomarkers into clinical practice.

## 1. Introduction

Ovarian cancer (OC) is one of the leading causes of cancer deaths in women. According to the Global Cancer Observatory GLOBOCAN 2020 database, OC accounted for 313,959 new cases (1.6% of all cancers) worldwide in 2020 with around 207,252 deaths (2.1% of all cancers) [1,2]. Moreover, according to the International Agency for Research on Cancer (IARC), the estimated number of global OC incidents for 2040 will be 434,184 new cases and 293,039 deaths, with respective increased incidence and death rates of 27.7% and 29.3% [3]. The majority of OC cases are of epithelial origin, which are named epithelial OC (EOC). According to the 5th edition of World Helath Organization classificaton, there are at least five subtypes of EOCs based on histomorphology, immunoprofile, and molecular pathogenesis: high-grade serous carcioma (HGSC), low-grade serous carcioma (LGSC), endometrioid carcinoma (EC), clear cell carcinoma (CCC), and mucinous carcinoma (MC) [4]. Among these, HGSC is the most common type (70%) and accounts for most deaths of EOCs. Approximately 75% of all women with EOCs are diagnosed when the cancer has spread into the peritoneal surface, corresponding to International Federation of Gynecology and Obstetrics (FIGO) stages IIIc and IV. The 5-year survival rate for patients with FIGO stage IIIc-IV EOC is <30%, and most deaths occur within the first 2 years after a diagnosis [5]. With the addition of the antiangiogenic agent bevacizumab and the poly (ADP-ribose) polymerase (PARP) inhibitors, there is only a modest improvement in progression-free survival (PFS) [6,7,8], however, there has been no significant improvement in overall survical (OS). A recently published phase 3 trial using maintanance PARPi olaparib therapy in paients with platinum-sensitive relapsed ovarian cancer carrying *BRCA1/2* mutation showed a median overall survival benefit of 12.9 months with a median follow-up perioid of about 65 months. However, a statistical significance was not accomplished which may be due to crossover and use of post-progresssion therapies [9] Thus, a comprehensive understanding of the mechanisms and molecular profiles is needed to improve clinical management and identify new therapeutic targets of EOC. In this review, we provide an updated overview of the different molecular profiles described for EOC and discuss their roles in current and future management of this malignancy.

## 2. The Molecular Landscape of EOC

EOCs represent a group of heterogeneous diseases with different precursor lesions, molecular alterations, and hence should be taken into consideration when choosing adjuvant therapeutic agents based on the current concept of targeted therapy. By integrated genomic analyses including exome sequencing, copy number analysis, mRNA expression, miRNA expression, and DNA methylation analysis, the molecular alterations of HGSC identified by The Cancer Genome Atlas (TCGA) [10] showed high frequency of *TP53* mutation (96%) and frequent alteration of homologous recombination related genes (51%) including *BRCA1/2* germline and somatic mutations (22%). Four molecular subtypes (immunoreactive, proliferative, differentiated, mesenchymal) were identified by gene expression analysis, and were demonstraed to have distinct clinical outcomes. LGSCs more frequently harbor *BRAF*, *KRAS*, or *NRAS* mutations [11]. A recent comprehensive genomic analysis and so far the largest genetic study of LGSCs [12] using whole exome sequencing and genome-wide copy number analysis demonstrated mutations of these key genes in the *RAS*/*RAF* pathway in 47% of cases, and up to 57.7% of cases had alterations in genes linked to this pathway. This study also identified mutations in putative novel driver genes including *USP9X*, *MACF1*, *ARID1A*, *NF2*, *DOT1L*, and *ASH1L*. Using Reactome pathway analysis, the top recurrently affected pathways include RAS signaling (22%), FGFR signaling (15%), MAPK signaling (15%), ErbB4 signaling (13%), chromatin organization (10%), and ubiquitination (10%). Molecular profiling by targeted massively parallel sequencing or whole genome sequencing, ECs were found to have frequent *CTNNB1*, *PIK3CA*, *PTEN*, *ARID1A*, *KRAS* mutations [13]. The molecular lanscape of ovarian EC is similar to that of the endometrial EC, although with mild varation in mutational frequencies. Akin to the molecular classification defined by TCGA for endometrial carcinoma [14], ovarian EC can also be classified into four molecular subtypes: 3% POLE (ultramutated), 19% MSI (microsatellite instability) (hypermutated), 17% copy-number high (serous-like) and 61% copy-number low (endometrioid). The signaling pathways frequently involved include WNT/β-catenin pathway, PI3K pathway, MAPK pathway, and SWI/SNF complex [13]. CCCs, similar to ECs with endometriosis as a possible precursor, may have mutations of *ARID1A* (around 50%), *PIK3CA* (around 50%), *TERT* (telomerase reverse transcriptase) promoter (5.7–16%), *PTEN* (2–13%), *KRAS* (4.7–20%), or amplifications of *AKT2* (8–26%), *MET* (24–37%), *ZNF217* (20–36%) as revealed by next generation sequencing of whole genome, whole exome, or targeted genes [15]. By whole genome sequencing, RNA sequencing, and copy number analysis, MCs, different from other types, most commonly have copy-number loss of *CDKN2A* (76%). Other frequently mutated genes include *KRAS* (64%) and *TP53* (64%) [16]. The distinctive genetic and epigenetic dysfunctions in different histological types of EOC involve kinases and transcription factors-associated pathways which promote the tumorigenesis and progression of EOC. In this section, we describe the major molecular pathways identified in EOC.

### 2.1. Mitogen-Activated Protein Kinase (MAPK)/Extracellular Signal-Regulated Kinase (ERK) Signaling Pathway

**Figure 1:**
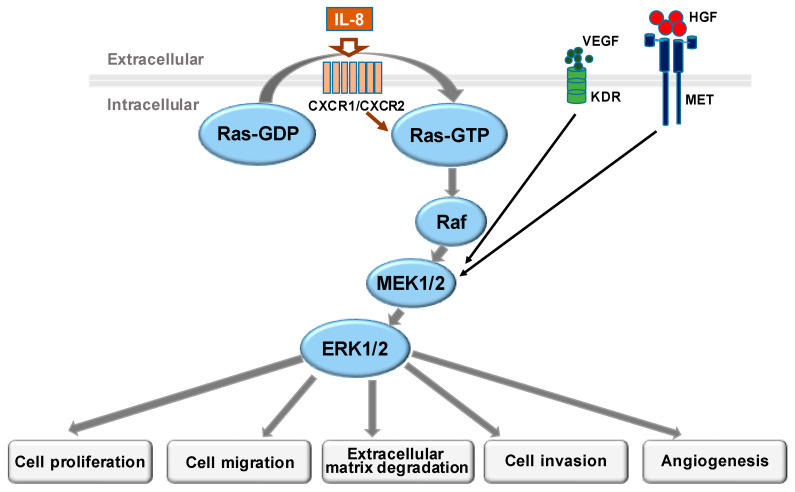
Role of mitogen-activated protein kinase (MAPK)/extracellular signal-regulated kinase (ERK) in epithelial ovarian cancer (EOC). The ERK/MAPK signaling pathway regulates tumorigenesis in EOC through cell proliferation, cell migration, extracellular matrix degradation, cell invasion, and angiogenesis. KDR, kinase insert domain receptor; MET, mesenchymal epithelial transition factor; CXCR, C-X-C motif chemokine receptor.

MAPK cascades are key signaling pathways that regulate many cellular processes, including proliferation, differentiation, apoptosis, and stress responses. The Ras/Raf/MAPK (MEK)/ERK pathway (Figure 1) is the most important and thoroughly studied signaling cascade among all MAPK signaling pathways, and it plays a crucial role in the cell signal transduction network and development of tumor cells [17,18]. ERK/MAPK signaling can be stimulated by various factors, such as cytokines, viruses, and oncogenes, and is activated in the following ways: (i) Ca^2+^ activation; (ii) receptor tyrosine kinase Ras activation; (iii) PKC-mediated activation; and (iv) G protein-coupled receptor activation [19]. Therefore, it is closely related to tumor formation. In EOC tumorigenesis, Hong et al. [20] found that expression levels of MAPK and ERK in EOC tissues were higher compared with those in adjacent normal tissues, while inhibition of the ERK/MAPK signaling pathway could restore tumor cells to a non-transformed state in vitro and in vivo, suggesting that increased activation of ERK/MAPK signaling may be closely associated with the progression of EOC. In addition, activation of ERK/MAPK signaling was also correlated with the characteristics of OC tumorigenesis, including (i) promoting cell proliferation via vascular endothelial growth factor (VEGF)-caused inhibition of apoptosis; (ii) increasing tumor invasion and metastasis by upregulating expression of matrix metalloproteinases (MMPs) that hydrolyse the extracellular matrix (ECM) in the tumor microenvironment (TME); and (iii) regulating expressions of transcription factors (such as hepatocyte growth factor (HGF), VEGF, and interleukin (IL)-8) that cause cytoskeletal deformation, and enhance tumor cell migration and tumor angiogenesis [21,22].

### 2.2. Phosphatidylinositol 3-Kinase (PI3K)/Protein Kinase B (AKT)/Mammalian Target of Rapamycin (mTOR)/Nuclear Factor (NF)-κB Signaling Pathways

**Figure 2:**
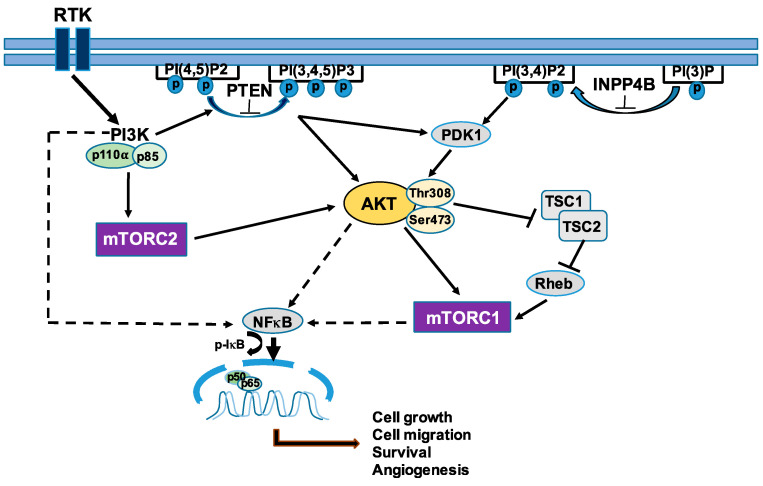
The phosphatidylinositol 3-kinase (PI3K)/AKT/mammalian target of rapamycin (mTOR)/nuclear factor-κ light chain enhancer of activated B cells (NF-κB) pathway. Activation of this pathway leads to enhanced cell growth, migration, survival, and angiogenesis. Indirect activation was shown by dashed arrows.

Hyperactivation of the PI3K/AKT/mTOR pathway occurs in nearly 70% of OCs and has been found to be the most frequently altered pathway in EOC [23] (Figure 2). This pathway plays multiple oncogenic roles including cell proliferation, survival, transcription regulation, protein synthesis, autophage, and angiogenesis [23]. In EOC, this pathway can be activated through several mechanisms including activating mutations of *PIK3CA* in CCC (30–40%), EnCA (12–20%) [24], mutations or amplifications of *PIK3CA* in HGSC (18%) [10]. Other subunits altered include mutation of PI3K p85 (*PIK3R1*) [25], mutations or amplifications of *AKT1*, *AKT2* [26,27], loss of *phosphatase and tensin homolog (PTEN)* through deletion, loss of function mutation, loss of heterozygosity, or epigenetic silencing [28], loss of inositol polyphosphate4-phosphatase type II (*INPP4B*) [29], and mutation of *mTOR* [30,31]. Among these subunits, PI3K p110α (*PIK3CA*) is the most frequently hyperactivated subunit within the PI3K pathway in EOC [23]. The increased activation of PI3K in EOC and its central role in several cancer-promoting pathways explain its implications in cancer progression including oncogenic transformation, cell proliferation, adhesion, and apoptosis, as well as multiple metabolic programming [23,32]. Moreover, the AKT/protein kinase B (PKB) family encompasses a group of serine threonine kinases, which act as oncogenes through mutation in the plekstrin homology (PH) domain or amplification, and they can be independently activated irrespective of the upstream PI3K signaling. In EOC, activation of AKT causes growth deregulation and strong resistance to apoptotic stimuli, conferring increased resistance to platinum, cisplatin, and paclitaxel [33], leading to uncontrolled tumor growth and cell invasion [34]. Additionally, mTOR is activated in about 50% of all HGSC [35], and it is known to be involved in cell growth, angiogenesis, evasion of cell death, and is thereby associated with poor prognoses [10,12].

An in silico analysis of PI3K/AKT/mTOR and NF-κB expressions, and correlation analyses of EOC TCGA samples using the Kaplan-Meier plot (http://kmplot.com/analysis, Accessed on 11 August 2012) [36] and gene expression profiling interactive analysis (GEPIA) web tools (http://gepia.cancer-pku.cn/index.html Accessed on 11 August 2012) [37] revealed that high expression of PI3K subunits are associated with poor survival. The complex crosstalk between the PI3K pathway and NF-κB results in decreased survival rates in EOC patients, enhanced aggressiveness, and chemoresistance, indicating that therapeutic targeting of PI3K/AKT/mTOR/NF-κB may be opportunities for clinical trials [38,39,40].

### 2.3. Janus Kinase (JAK)/Signal Transducer and Activator of Transcription (STAT) Signaling Pathway

**Figure 3:**
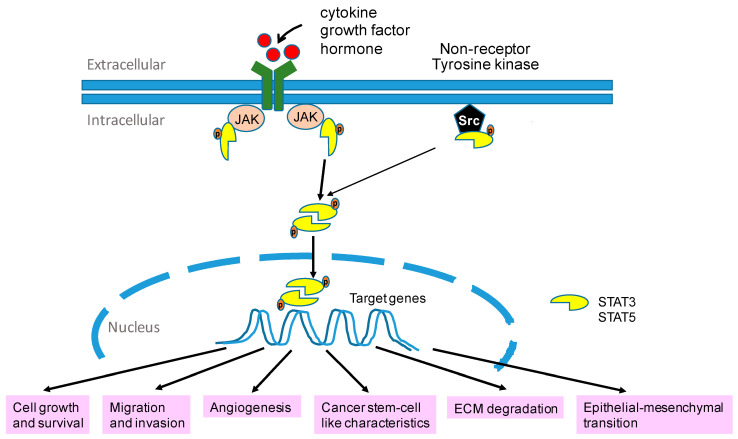
Activator of transcription 3 (STAT3)/5 signaling pathway. Activation of STAT3/5 promotes target gene expression, which contributes to tumor growth, survival, invasion, angiogenesis, stem cell-like characteristics, extracellular matrix (ECM) degradation and epithelial-mesenchymal transition.

Activation of the STAT3/STAT5 pathway regulates a variety of cellular processes, such as tumor cell growth, survival, invasion, cancer stem cell-like characteristics, angiogenesis, drug-resistance, degradation of extracellular matrix, and epithelial-mesnechymal transition (Figure 3), which have significant correlations with reduced survival of recurrent EOC [41,42]. Activation of STAT3 had been found in ovarian cancer cell lines [43] and ovarian tumor tissues especially high-grade carcinomas including HGSC and CCC [44]. Activaton of STAT3/STAT5 is mainly through tyrosine phosphorylation, by various stimuli such as cytokines, growth factors, hormones, and oxidative stress. Upon activatioin, dimerization and nuclear translocation result in transcription of target genes [41,42]. Alpinetin, a natural flavonoid, inhibits cell migration through downregulating matrix metallopeptidase (MMP)-2 and MMP-9 via suppression of STAT3 signaling in EOC [45]. It was also reported that constitutively activated STAT3 is involved in the epithelial-mesenchymal transition (EMT) of EOC by upregulating vimentin, N-cadherin, and IL-6 in STAT3-activated cells. Moreover, activation of STAT3 has strong correlations with elevation of Bcl-xL, cyclin D1, and c-myc, thereby promoting cell proliferation and survival [46]. Notably, STAT3 plays a vital role in regulating hypoxia-inducible factor (HIF)-1α which is a key modulator of angiogenesis [47], thus facilitating EOC angiogenesis. Furthermore, studies also demonstrated that STAT3 is correlated with expressions of stemness markers, including c-myc, Nanog, aldehyde dehydrogenase 1 family, member A1 (ALDH1A1), cluster of differentiation 24 (CD24), and β-catenin, which were found to be increased in OC spheroids [48] and correlated with chemoresistance [49].

### 2.4. Wnt/β-Catenin Pathway

**Figure 4:**
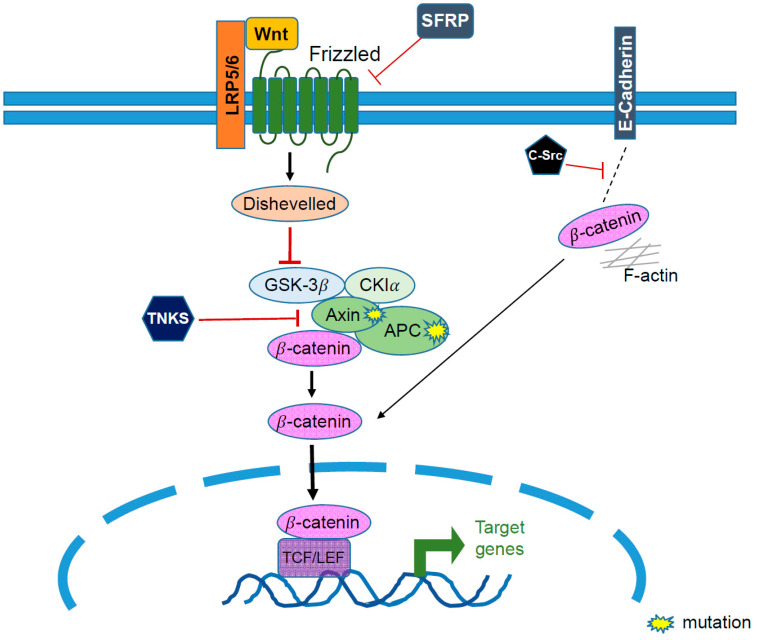
The aberrant activation of Wnt/β-catenin pathway contribute to tumorigenesis in EOC. LRP, LDL receptor related protein; SFRP, secreted frizzled related protein; TNKS, tankyrase; AXIN, axis inhibition protein; APC, adenomatous polyposis coli.

Dysregulation of the Wnt/β-catenin pathway (Figure 4), which leads to hyperactivation of β-catenin, was reported to promote cancer stem cell (CSC) self-renewal, metastasis, chemoresistance, angiogenesis, and immune suppression in all subtypes of EOC [50]. Specifically, mutation of β-catenin encoding gene *CTNNB1* is frequently found in EC (up to 54%) [51]. Other rare mutations of the β-catenin destruction complex include mutations of AXIN in EC [51], and mutation of APC in MC [52]. Furthermore, higher activity of β-catenin through several mechanisms has been found in EOC, especially HGSC [50]. Accumulating evidence showed that modulation of β-catenin activity, such as microRNA (miR)-1207, suppression of suppression of secreted frizzled-related protein 1 (SFRP1) and axis inhibition protein 2 (AXIN2) (negative regulators of the Wnt/β-catenin pathway), activation of β-catenin signaling, and promotion of expressions of CSC markers promote stemness and chemoresistance in EOC [53,54]. Moreover, Wnt/β-catenin signaling is involved in the remodeling of the extracellular tumor matrix such as MMP-2, -7, -9, and E-cadherin, suggesting enhancement of the EMT/metastasis and angiogenesis in ovarian tumors [55]. Additionally, EOC was reported to evade the immune system through multiple mechanisms, including the recruitment of regulatory T cells (Treg) and promotion of T-cell apoptosis via programmed death ligand-1 (PD-L1) [56]. In addition, IL-10 and indolamine 2,3-dioxygenase (IDO) were reported to promote immune evasion by ovarian tumor-associated macrophages [57], thereby promoting EOC progression.

## 3. Molecular Markers for Surveillance of EOCs

Early diagnosis of EOC remains an important unmet medical need. To date, two-stage detection strategies have been utilized using biomarker Cancer Antigen 125 (CA125, also known as MUC16) [58] and/or human epididymis protein 4 (HE4, also known as WFDC2) levels in blood, and subsequent transvaginal sonography (TVS) examination [59]. However, their sensitivity and specificity are not sufficient enough to detect EOC at the early stage which has a strong correlation with the patient’s survival rate. To improve the initial stage of clinical screening, a combination of CA125 and HE4 has been used to become multi-parametric assays such as the OVA1 test, risk of ovarian malignancy algorithm (ROMA) test, and OVA2 (or OVERA) which are the clinically approved blood-based biomarkers for EOC risk stratification. OVA1 assay was approved by the FDA in 2009 and evaluates combined data from imaging, menopausal status, and five serum protein biomarkers including second-generation CA125-II, apolipoprotein A1, transthyretin, transferrin, and β2-macroglobulin [60]. When OVA1 was used along with the physical assessment of the patient, an improvement of sensitivity at 96% and specificity at 35% were observed [61]. ROMA is a screening test which was approved by the FDA in 2011 for predicting the risk of EOC in patients with pelvic masses [62]. It is a combination of CA125, HE4 levels in blood and the menopausal status of the subject. ROMA produced a sensitivity of 100% and a specificity of 74.2% in premenopausal patients, while the postmenopausal had a sensitivity of 92.3% and a specificity of 76%, respectively [62,63]. OVERA, the second-generation test for OVA1, is a multi-parametric test combining CA125-II, HE4, apoliprotein A1, transferrin, and follicle-stimulating hormone for screening patients with pelvic masses. Compared to OVA1, OVERA test was reported to improve its sensitivity and specificity of 91% and 69%, respectively, therefore was approved by FDA in 2016 [64]. Obviously, no single biomarker can accurately detect EOC at an early stage, thereby new molecular techniques which allow identify potential clinical epigenetic signatures are important in detection of OC carcinogenesis. In Table 1, we summarize the clinical utility of biomarkers and list their applications as well as their sensitivity/specificity for clinical prediction of EOC (Table 1).

Recent studies showed steady improvements in diagnostic accuracies using circulating cell-free (cf)DNA resulting from genetic mutations, copy number alerations, allelic imbalance, and promoter methylation in tumors [65]. Using subgroup analyses and meta-regression analyses of systematic reviews suggested that epigenetic markers (i.e., epigenetic DNA modifications, methylation, and alterations) are particularly more effective over other quantitative detection methods of cell-free (cf)DNA and RNA concentrations or chromosomal instability [65,66]. Many susceptibility genes in hereditary ovarian carcinomas are known to alter homologous recombination (HR)/DNA repair pathways [67,68], and recent genomic/epigenomic studies indicated that EOC represents a genetically heterogeneous and complex group of diseases [67,68,69]. The genetic heterogeneity may reflect exerted clonal selection in the progression (i.e., chemoresistance and metastatic capacity) of EOCs. This review discusses genomic advances in EOC, and focuses on translating these genomic (both somatic and germline) alterations (as useful biomarkers) into clinical practice.

Approximately 15~20% of EOCs occur in a familial context with a high penetrant autosomal dominant genetic predisposition. To increase EOC patient survival, application of biomarkers for early diagnosis/detection and risk factor prediction, including genomic/epigenomic variants, copy number aberrations (CNAs), and DNA methylation in EOC surveillance are promising [66,70]. Moreover, due to rapid developments in DNA sequencing technology, various novel germline mutations have also been identified in familial EOC cases and in patients with early-onset EOC. These selected biomarkers and their clinical significance in EOCs are described in Table 2.

## 4. Molecular Markers to Guide Targeted Treatment of Advanced EOCs

### 4.1. Translational Insights into Transforming Genomic Alterations into Clinical Practice

In addition to using biomarkers as diagnostic/detection tools to improve surveillance in EOCs, biomarkers are also helpful as genetic predictors of treatment responses in EOC patients. Recent studies showed that several genetic predictors (Table 2) of treatment outcomes in EOC patients were identified from new high-throughput genomic techniques, including genome-wide association studies (GWASs), whole-exome sequencing (WES), whole-genome sequencing (WGS), DNA CNAs, and epigenetic alterations. For example, in carboplatin- and paclitaxel-based chemotherapy in 1244 patients with serous OC, a GWAS analysis identified that minor alleles of two single-nucleotide polymorphisms (SNPs) (rs7874043 and rs72700653) were associated with poor PFS [110]. Furthermore, genetic variations in OCs are complex and differ among different histological types. Therefore, comprehensive genetic analysis and pathway annotation may provide better chance for the personalization of therapy, and patients with concurrent alterations in multiple pathways appear to havea high potential for combinations of targeted therapies [111].

In addition, WES and exome array-captured sequencing can be used to discover low-frequency variants in individuals with familial high-penetrant diseases and those with certain complex quantitative traits. Most patients with HGSC initially benefit from platinum- and taxol-based chemotherapy, but progressive chemoresistance occurs and results in tumor recurrence and eventual metastasis. Little is known about the mechanisms of chemoresistance. One study applied WES and SNP profiling of 31 paired EOC tissues before and after first-line platinum-based chemotherapy [112]. They found frequent homologous recombination (HR)-deficiencies [i.e., loss-of-heterozygosity (LOH) and mutations in HR genes] in primary tumors, relapsed tumors, and tumors resistant to second-line platinum, implicating persistent HR deficiencies and qualification for second-line poly(adenosine diphosphate [ADP]-ribose) polymerase (PARP) inhibitors (e.g., olaparib and rucaparib) treatment in recurrent/chemoresistant tumors. Increased CNAs of several genes, including *MDS1 and EV11 complex locus(MECOM)*, *G1/S-specific cyclin-E1* (*CCNE1)* [112], and *Erb-B2 receptor tyrosine kinase 2* (*ERBB2)*, were discovered in recurrent tumors. Additionally, genomic instability is a hallmark of cancer, and genomic aberrations in the form of DNA CNAs are important in EOC tumorigenesis [67,113,114], and specific CNAs are potential biomarkers for chemoresistance, tumor relapse, and survival in EOCs (Table 3).

### 4.2. Targeted Agents and Profiling Utilization Registry

The heterogeneous characteristic of EOC and its direct relationship with treatment failure are well documented [133]; thus, the broad mutational landscape of EOC, coupled with increasing availability of sequencing technologies, implies the application of precision-based biomarkers in EOC diagnostics and surveillance, including the use of circulating tumor DNA [134]. Therefore, comprehensive molecular profiling of EOC could lead to the development of new personalized or precision medicine in EOC. According to this scenario, current clinical oncology trials across a range of tumors using biomarker-driven designs were undertaken, including the National Cancer Institute’s Molecular Analysis for Therapy Choice (NCI-MATCH) trial [135,136], the American Society of Clinical Oncology (ASCO) Targeted Agent and Profiling Utilization Registry (TAPUR) study, and the European Organization for Research and Treatment of Cancer-Screening Patients for Efficient Clinical Trial Access (EORTC-SPECTA) program. Among these, the novel phase II NCI-MATCH trial was initiated in August 2015 to investigate agents (in addition to approved agents) of matching targeted therapy to molecular/genomic profiles. The trial is running under ClinicalTrials.gov identifier NCT02465060, where updated information can be obtained. Furthermore, the TAPUR study is an ongoing, nonrandomized, multicenter clinical trial that opened in 2016 [137]. This trial is testing the use of drugs already approved by the US Food and Drug Administration (FDA) that target a specific tumor mutation in individuals with advanced cancer outside of the drug’s approved indication. Patients are treated according to their molecular profile regardless of the tissue origin or cancer type as shown in Table 4.

## 5. Conclusions

EOC causes substantial morbidity and mortality in the developed world. A number of clinical features are known to affect PFS and OS rates in EOC, including the disease stage, tumor grade, chemoresistance, and recurrence. Current concepts in treating EOC are focused on new therapies (e.g., PARP inhibitors) as well as molecular testing. At present, only the *BRCA* status is routinely used clinically, with germline *BRCA1* and *BRCA2* genetic testing now in place at a number of centers as a biomarker for the use of PARP inhibitor therapy. The benefits of genetic testing for EOC patients and their family members can guide personalized treatment decisions, while also potentially preventing disease in others who carry inherited gene mutations. Therefore, genomic defects in homologous recombination DNA repair (HRR) pathway components remain an area of great interest, which is believed to benefit and improve clinical therapeutic outcomes. Although molecular markers/profiling for diagnosis, prognosis prediction, surveillance, and treatment are becoming more important as knowledge of the molecular mechanisms of EOC increases, molecular testing still faces several challenges, which needs to be addressed before broad implementation into clinical practice can be achieved. There are currently several ongoing clinical trials, which are investigating new targeted treatments for advanced EOCs. This will allow researchers and clinicians to work with useful diagnostic and therapeutic tools to combat advanced EOCs. The keys to success are molecular testing and personalized medicine, to obtain flexibility and fit the treatment for each patient with a unique EOC type.

## Figures and Tables

**Table 1:** List of selective techniques and biomarkers for EOC detection.

Technique	Biomarkers	Source	Sensitivity (SN)/Specificity (SP)	References
Enzyme-linked immunosorbent assays (ELISA) or chemiluminescence immunoassays	Protemics such as: CA125, HE4, Mesothelin, ApoA1, and Osteopontin	Serum, Plasma, Urine	SN: 84% SP: 98.5%	[71,72]
Liquid chromatography tandem mass spectrometry (LC-MS/MS)	Protemics such as: CA125, HE4, Mesothelin, ApoA1, and Osteopontin	Serum, Plasma, Urine	SN: 89% SP: 92%	[72,73,74]
Methylation-specific PCR (MSP)	Hypermethylated genes such as: p16, DAPK, PTEN, APC, BRCA1	Tumor tissue, serum, blood DNA, peritoneal fluid	SN: 82% (for serum), 54% (for blood or tissue DNA), 93% (for peritoneal fluid) SP: 100% (all tumor stages)	[75,76]
Microarray based multiplex assay (MethDet56 technique)	10-gene panel (combination of BRCA1, HIC1, PAX5, PGR, THBS1)	Plasma, circulating tumor DNA (ctDNA)	Presence of malignancy SN: 61% SP: 85%	[77]
Targeted ultra-high coverage bisulfite sequencing	3-DNA-methylation-serum-marker panel	Serum cell free DNA (cfDNA)	Pre-chemotherapy (SN: 41.4%, SP: 90.7%); Post-chemotherapy (SN: 78%, SP: 86%)	[78]
Quantitative RT-PCR (qRT-PCR); gene expression array	Dicer, Drosha mRNA	Tumor tissue, cell lines	Advanced tumor stage	[79]
Direct Sanger sequencing; Next-generation sequencing (NGS)	miRNAs: miR-124-1, -127, -132 and -339.	Tumor tissue, ctDNA	EOC metastasis (including peritoneal macro-metastases)	[80]
miRNA microarray; Stem-loop qRT-PCR	miRNAs: let-7i	Tumor tissue, cell lines	Associated with chemoresistance and progression-free survival	[81]
Northern blots; In-situ hybridization	miRNAs: miR-34a, miR-30c	Tumor tissue, cell lines	Associated with development of chemoresistance, motility, and invasion.	[82]
MethyLight assay	Methylation of miRNAs: miR-34a	Tumor tissue	Inversely associated with grade, p53 mutation, and dualistic tumor type.	[83]
Small RNA sequencing	miRNAs: hsa-miR-135, 150, -340, 625, 1908, and -1275	Tumor tissue	Associated with survival	[84]
Luciferase reporter assay, Western Blot, and Cytotoxicity assay	miRNAs: miR-9	Tumor tissue and cell lines	Associated with chemoresistance	[85]
Ultra-performance liquid chromatography (UPLC); matrix-assisted laser desorption/ionization time-of-flight (MALDI-TOF); and capillary electrophoresis (CE)	Glycomics: N-Glycan, glycopeptide, and glycoprotein.	Serum, tumor tissue	SN: 97% SP: 98.4%	[86,87]

**Table 2:** Summary of selected genetic alterations and their clinical significance in ovarian carcinomas.

Major Genetic Alteration	Clinical Implications	References
**Detection/diagnosis** Germline mutations in *BRCA1* or *BRCA2* Novel germline mutations in *BARD1*, *BRIP1*, *PALB2*, *RAD50*, *RAD51C*, *RAD51D*, *TP53*, *ASXL1*, *MAP3K1*, *SETD2*, etc. Germline mutations in *SMARCA4* (*BRG1*) Mutations of *PIK3CA*, *RB1*, and *MED1* in plasma of EOC patients following therapy Germline mutations in mismatched repair (MMR) genes (*MSH2*, *MSH6*, *MLH1*, and *PMS2*)	Increased risk of EOC; surveillance for early EOC. A subset of familial EOCs with high and moderate penetrance or a moderate EOC susceptibility that may warrant their use in routine clinical genetic testing. Improvements in genetic counseling and early detection for SCCOHT. Applicable for monitoring EOC patients with high systemic tumor burdens, metastasis, and therapeutic responses. An 8%~10% risk of EOC (Lynch syndrome); routine clinical surveillance for early OC.	[88,89,90,91,92,93,94,95]
**Risk assessment** Rs7651446(3q25), rs9303542(17q21), rs11782652(8q21), rs1243180(10p12), and rs757210(17q12) Rs8170 and rs2363956 at 19p13.11 Rs3814113(9p22.2) Rs752590(2q13), rs711830(2q31.1), and rs688187(19q13.2) Rs3814113(9p22.2)	Predicting EOC risk. Predicting survival and genome-wide serous OC risks. EOC risks, strongest for serous OC. Risk associations with mucinous OC. A reduced EOC risk in *BRCA1*/*BRCA2* mutation carriers.	[96,97,98,99,100,101]
**Oncogenes** *WNT6* (wingless-type MMTV integration site family, member 6) *COL23A1* (collagen, type XIII, alpha 1) *C2CD4D* (C2 calcium-dependent domain containing 4D)	Involved in cell proliferation, differentiation, and adhesion. Likely involved in cell adhesion. Unknown.	[78]
**Tumor suppressors** *RASSF1A* (Ras association domain-containing protein 1) *OPCML* (opioid-binding protein/cell adhesion molecule-like gene) *DAPK* (death-associated protein kinase 1) *APC* (adenomatous polyposis coli) *TFP12* (tissue factor pathway inhibitor 2) *PAX1,5* (paired box 1, 5) *PTEN* (phosphatase and tensin homolog)	Modulates multiple apoptotic and cell-cycle checkpoint functions. Involved in cell adhesion and cell-cell recognition. Involved in multiple cell death-associated signaling pathways. Controls cell division, motility, and adhesion in Wnt/β-catenin signaling pathway. Involved in regulating extracellular matrix digestion and remodeling. Involved in cell development. Regulates cell proliferation.	[75,77,102,103,104,105,106,107,108,109]

Abbreviations: *BRCA1/2*, breast cancer type 1/2 susceptibility protein; *BARD1*, *BRCA1*-associated RING domain 1; *BRIP1*, *BRCA1* interacting protein C-terminal helicase 1; *PALB2*, partner and localizer of *BRCA2*; *SETD2*, SET domain containing 2; *SMARCA4*, SWI/SNF-related, matrix-associated, actin-dependent regulator of chromatin, subfamily A, member 4; SCCOHT, small cell carcinoma of hypercalcemic type.

**Table 3:** Summary of selected genetic alterations and therapeutic monitoring in ovarian carcinomas.

Major Genetic Alterations	Clinical Implications	References.
**Chemotherapeutic response/prognosis evaluation** Germline/somatic mutations in *BRCA1*, *BRCA2*, and other genes in the HR pathway Rs7874043 in TTC39B Rs4910232(11p15.3), rs2549714(16q23), and rs6674079(1q22) Rs1649942 Mutations of eight members of the *ADAMTS* family Gains on 1q, 5q14~q23, and 13q21~q32, and losses of 8p and 9q Loss of 13q32.1 and 8p21.1 Gains in 9p13.2-13.1, 9q21.2-21.32, 9q22.2-22.31, 9q22.32-22.33, and 9q33.1-34.11 Losses of 4p, 4q31.1-qter, 5q12-q22, 8p, 16q, and X*CCNE1* amplification High-level amplification at 8q24 and loss of 5q Gain in 5p; gain in 1p and loss of 5q *Met* amplification in ovarian clear cell carcinoma	(1) Predictive of platinum sensitivity and longer survival in women with HGSC; (2) benefits from PARP inhibitors. The minor allele is strongly associated with poor PFS in patients with HGSC following first-line chemotherapy. These rare alleles were significantly associated with poorer outcomes in EOC patients who underwent first-line treatment of cytoreductive surgery and chemotherapy. Associated with decreased PFS and poorer OS in EOC patients with carboplatin-based chemotherapy. Associated with significantly higher chemotherapy sensitivity and better OS and PFS in HGSC. Clinical carboplatin resistance. Predictive markers of chemoresistant serous carcinoma. Potential predictive markers of docetaxel/carboplatin resistance. Poor survival in stage III EOC Poor prognosis in postoperative EOCs. Favorable prognosis for serous carcinoma. A higher risk of recurrence; significant decrease in recurrence. Worse survival.	[67,110,115,116,117,118,119,120,121,122,123,124,125,126,127]
**Targeted therapy/individualized therapy** Various molecular subtypes of HGSC signatures associated with survival Few point mutations in LGSCs and borderline tumors Recurrent mutations in *ELF3*, *RNF43*, *ERBB3*, and *KLF5* in mucinous OC Heterogeneity in the genome of HGSC under selective pressure of chemotherapy *PPM1D* amplification *HER2* amplification	Provides an opportunity to improve HGSC outcomes through subtype-stratified care. Targeted therapeutic agents against *BRAF* and *KRAS* might be particularly effective for recurrent inoperable cases. Potential novel targeted therapy for some high-grade mucinous carcinomas. Overcoming resistance to conventional chemotherapy will require a diversity of approaches, such as use of new inhibitors of MDR1 and PARP. A potential therapeutic target for a subgroup of ovarian CCCs A potential therapeutic target.	[10,67,128,129,130,131,132]

Abbreviations: PARP, poly(adenosine diphosphate-ribose) polymerase; *CCNE1*, cyclin E1; RNF43, ring finger protein 43; *ERBB3*, Erb-B2 receptor tyrosine kinase 3; *KLF5*, Krüppel-like factor 5; *PPM1D*, protein phosphatase magnesium-dependent 1δ; *HER2*, human epidermal growth factor receptor 2; PFS, progression-free survival; *BRAF*, v-raf murine sarcoma viral oncogene homolog B1; MDR1, multidrug resistance 1.

**Table 4:** Molecular profiling boundaries—biomarker-targeted therapy matches.

Targeted Mutation	Drug
**NCI-MATCH trial: NCT02465060 ^a^**	
• *EGFR*, or *ERBB2*-activating mutation	Afatinib
• *BRCA1* or *BRCA2* mutations; Wee1 inhibitor	Adavosertib (AZD1775)
• *AKT*1, 2, or 3 mutations	Capivasertib (AZD 5363)
• *PIK3CA* or *PTEN* mutation, *PTEN* loss	Taselisib, GSK2636771 (a PI3Kβ inhibitor)
• *MET* amplification or exon 14 skipping; *ALK* or *ROS1* translocation	Crizotinib
• *BRAF* V600E/V600R/V600K/V600D mutation	Dabrafenib + trametinib
• *FGFR* mutation, fusion, or amplification	Erdafitinib (AZD4547)
• Loss of *MLH1*, *MSH2* or MMRd	Nivolumab
• *CDK4*, *CDK6* or *CCND1* amplification and/or Rb protein loss	Palbociclib
• *TSC1* or *TSC2* mutation, or *mTOR* mutation	Sapanisertib (MLN0128)
• *BRAF* fusion, *BRAF* non-V600, or *NF1* mutation	Trametinib
• *SMOIPTCH1* mutation	Vismodegib
**TAPUR trial: NCT02693535 ^b^**	
• *VEGF* mutation, amplification, or overexpression	Axitinib
• *ALK*, *ROS1*, or *MET* mutations	Crizotinib
• *KRAS*, *NRAS*, and *BRAF* (all wild-type)	Cetuximab
• *BRCA1*/*BRCA2*-inactivating mutations; *ATM* mutations/deletions	Olaparib
• MSI-high, high TML, and others	Nivolumab and ipilimumab
• *CDKN2A*, *CDK4*, and *CDK6* amplifications	Palbociclib
• *VEGFR1*, *VEGFR2*, *VEGFR3*, *PDGFRB*, *RET*, *KIT*, *RAF-1*, and *BRAF* mutations/amplifications	Regorafenib
• *PDGFR*, *VEGFR*, and *CSF1R* mutations/amplifications	Sunitinib
• *mTOR* and *TSC1/2* mutations	Temsirolimus, sapanisertib (MLN0128)
• *ERBB2* amplifications	Trastuzumab and pertuzumab
• *BRAF* V600E mutations	Vemurafenib and cobimetinib

Abbreviations: MMRd: mismatch repair deficiency; MSI, microsatellite instability; TML, tumor mutation load; *ALK*, anaplastic lymphoma kinase; *FGFR*, fibroblast growth factor receptor; *MLH1/2*, MutL homolog 1/2; *TSC1/2*, tuberous sclerosis complex 1/2; *VEGFR*, vascular endothelial growth factor; *PDGFRB*, platelet derived growth factor receptor-β; *CSF1R*, colony stimulating factor 1 receptor. ^a^ NCI-MATCH trial: Targeted Therapy Directed by Genetic Testing in Treating Patients With Advanced Refractory Solid Tumors, Lymphomas, or Multiple Myeloma. Matches are as listed on clinicaltrials.gov/ct2/show/NCT02465060 (accessed on 7 June 2021). ^b^ The American Society of Clinical Oncology’s TAPUR trial: Testing the Use of US Food and Drug Administration-Approved Drugs That Target a Specific Abnormality in a Tumor Gene in People with Advanced Stage Cancer. Matches are as listed on clinicaltrials.gov/ct2/show/NCT02693535 (accessed on 7 June 2021).
